# Immune Checkpoint Inhibitor–Related Myositis and Associated Triad Overlap Syndrome

**DOI:** 10.1002/acr.80015

**Published:** 2026-02-18

**Authors:** Selene Rubino, Grant W. Cannon, Brian C. Sauer, Jorge Rojas, Gregory J. Stoddard, Gary A. Kunkel, Jessica A. Walsh, Bryant R. England, Joshua F. Baker, Tawnie J. Braaten

**Affiliations:** ^1^ Mayo Clinic Rochester Minnesota; ^2^ University of Utah Salt Lake City; ^3^ VA Salt Lake City Health Care System Salt Lake City Utah; ^4^ VA Puget Sound Health Care System Seattle Washington; ^5^ VA Nebraska‐Western Iowa Health Care System Omaha Nebraska; ^6^ University of Nebraska Medical Center Omaha; ^7^ Corporal Michael J. Crescenz VA Medical Center Philadelphia Pennsylvania; ^8^ University of Pennsylvania Philadelphia

## Abstract

**Objective:**

Immune checkpoint inhibitor (ICI) myositis is a rare but a highly morbid condition, particularly with the ICI myositis triad syndrome of myositis, myocarditis, and myasthenia gravis. We report the clinical characteristics of ICI myositis and all‐cause mortality in these patients.

**Methods:**

We performed a national retrospective cohort study of US veterans treated with an ICI between June 2011 and February 2022. Cases were identified by elevated creatinine kinase (CK) following ICI therapy and/or “myositis” in notes. Chart review determined if candidates had ICI‐associated myositis and were subsequently classified as isolated ICI myositis or ICI triad syndrome.

**Results:**

Of 29,296 veterans treated with ICIs, 68 (0.2%) ICI‐induced myositis cases were identified, including 23 patients with ICI triad syndrome and 45 patients with isolated ICI myositis. Median survival duration for ICI triad syndrome was 2.5 (95% confidence interval [CI] 1.7–18.5) months in comparison to 20.4 (95% CI 6.2–60) months for isolated ICI myositis and 12.6 (95% CI 12.3–12.8) months median survival for patients treated with ICI without myositis. Patients with ICI triad syndrome had 60.0% mortality during the six months following initiation of ICI treatment, in comparison to 30.2% mortality in isolated ICI myositis and 31.5% mortality in patients without myositis. Six months after the first ICI treatment, subsequent mortality trends were similar in the three groups.

**Conclusion:**

ICI triad syndrome is associated with higher overall mortality and shortened median survival times during the first six months after initial ICI therapy, whereas isolated ICI myositis is associated with similar mortality outcomes compared to patients treated with ICI who did not develop myositis.

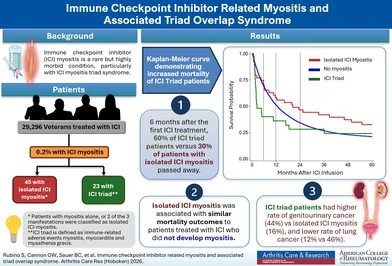

## INTRODUCTION

Since the introduction of the first immune checkpoint inhibitor (ICI), ipilimumab, in 2011, there has been a considerable expansion in number of ICIs and indications for ICIs.^1^ The number of adverse events of ICIs have also increased during the expanded use of these medications. Although myositis is a rare complication of ICI therapy, representing 0.6% of all immune‐related adverse events (irAEs) in pharmacovigilance databases,^2^, ^3^ there is considerable morbidity and significant mortality associated with its development. In 2020, Aldrich et al reported increased mortality in a subgroup of patients with ICI myositis who developed myocarditis and myasthenia gravis in addition to myositis following immunotherapy,^4^ termed ICI myositis triad syndrome. In line with findings of Aldrich et al, subsequent retrospective cohort studies measured the overall mortality of ICI myositis triad to be between 42% and 51%.^5^, ^6^ Among patients with ICI myositis who died, those who had overlapping myocarditis or myasthenia gravis–like features were more likely to die of myositis irAE than of the progression of cancer.^7^ The purpose of the current study was to evaluate the clinical features and outcomes of patients within the Veterans Health Administration (VHA) who developed ICI‐induced myositis and ICI myositis triad compared to patients treated with ICI who did not develop myositis.

**SIGNIFICANCE & INNOVATIONS**

- Two immune checkpoint inhibitor (ICI)–associated myositis phenotypes were observed: ICI myositis triad syndrome and isolated ICI myositis.
- ICI myositis triad syndrome is typically a severe acute illness with significant risk for death shortly after initiation of ICI treatment.
- Isolated ICI myositis is a less severe disease with less mortality than ICI myositis triad syndrome with outcomes generally similar to patients treated with ICI who do not develop myositis.

## PATIENTS AND METHODS

### Study design and data resources

We performed a national retrospective cohort study of veterans enrolled in VHA who underwent ICI therapy between June 6, 2011, and February 14, 2023. Electronic health record (EHR) data from the VA Corporate Data Warehouse, including demographic data, clinical notes, pharmacy data, and laboratory results, were extracted and analyzed in these patients.^8^ The VA Central Cancer Registry data (Oncology Raw Domain) provided information on cancer diagnoses, and mortality was obtained from the VHA Death Ascertainment File.^9^

### Case identification and classification

Briefly, patients were selected for chart review if they had one of the following:

1. A creatinine kinase (CK) level raised above 10 times the upper limit of normal (ULN) within six months of ICI administration
2. A CK level raised above 1 × ULN within six months of ICI administration and whose EHR included the term “myositis” in any discharge summary after first ICI administration
3. A CK level raised above 1 × ULN and the term “myositis” within progress notes within six months after the first ICI treatment

**Figure 1:**
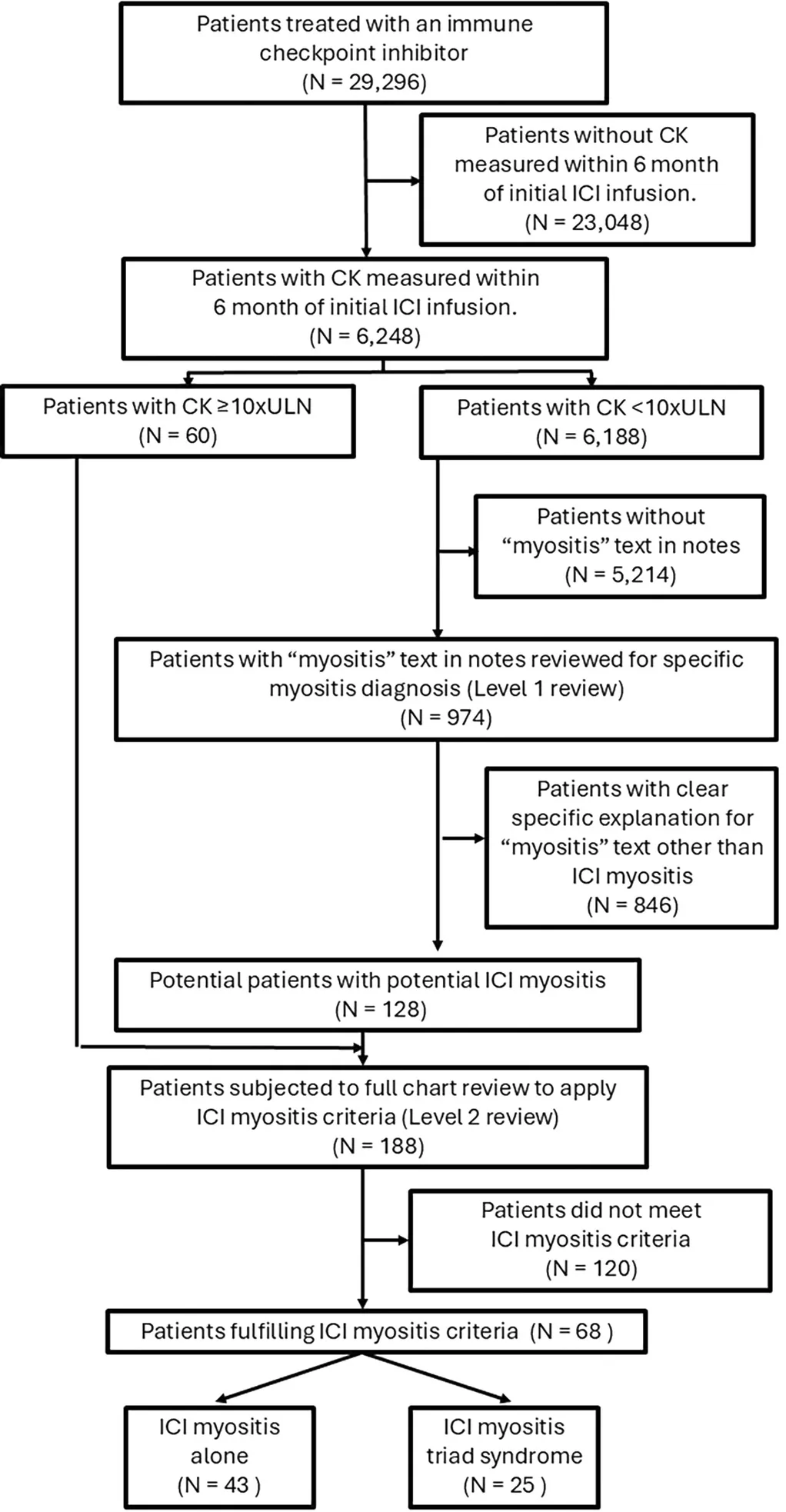
Flowchart outlining study selection and case categorization. CK, creatinine kinase; ICI, immune checkpoint inhibitor; ULN, upper limit of normal.

Our process for identifying patients with ICI myositis is presented in Figure 1. All patients treated with ICI with CK measured within six months of first ICI infusion were identified. We then searched for the text term “myositis” in clinic notes and discharge summaries, identifying patients with high levels of CK and *International Classification of Diseases* (ICD) codes for idiopathic inflammatory myositis. The review of text notes involved two levels of review. The level one review was conducted by GWC and involved reading notes to determine if a specific explanation for the “myositis” term other than ICI myositis was present. The majority of these conditions included statin‐induced myositis, baseline prior inflammatory myositis, the myositis term included in a problem list without evidence of any active muscle disorder, and negation statements such as “myositis absent.” Cases considered to potentially be ICI myositis were subjected to level two review by SR. This level two review assessed the entire medical record in addition to clinical notes and included assessment of consultations, imaging, and laboratory testing to determine if the patient met the ICI myositis criteria listed below. Cases in which there were any concerns about the diagnosis were adjudicated by SR and TJB.

In addition to the review of all patients with measured CK and “myositis” text in clinical notes, all patients with one or more CK level ≥10 × ULN were subjected to level one review. A preliminary review of patients with CK levels elevated between 2 × and 10 × ULN and CK levels elevated above 10 × ULN showed ICI myositis in 30% (3 of 10) and 42% (8 of 19) of patients, respectively. The 20 patients with “myositis” text notes and CK levels >10 × ULN were subjected to both level one and level two review. Of these 20 patients with both CK levels >10 × ULN and “myositis” text in clinic notes, 19 (95%) were classified as having ICI myositis on level two review. Thus, 10 × ULN was a value at which individual chart review should be considered for all patients with this level of CK elevation. ICD codes for idiopathic inflammatory myositis were associated with note text and not found to be useful for identifying ICI myositis. Of the 25 cases with idiopathic inflammatory myositis codes, 20 (80%) were identified by the screening of “myositis” text in notes, and a review of the other 5 cases did not show ICI myositis.

Patients were classified as having ICI‐induced myositis if they had: (1) a confirmed provider diagnosis of ICI‐induced myositis in the medical record by chart review, (2) an elevated CK level above the ULN after ICI therapy, and (3) no other explanation for the elevated CK level other than ICI‐induced myositis. Patients meeting ICI‐induced myositis criteria were further classified as ICI triad or isolated ICI myositis. The category “ICI triad syndrome” denotes patients who developed myositis, myasthenia gravis, and myocarditis at any point after the ICI was first administered. Myocarditis was defined as “possible” or “probable” myocarditis using the criteria proposed by Bonaca et al.^10^ Myasthenia gravis was determined by symptoms of ptosis, diplopia, and/or respiratory failure with associated decline in negative inspiratory force (dysphagia was excluded due to association with myositis). Patients with ICI myositis on chart review who did not develop all three manifestations of ICI triad were classified as “isolated ICI myositis,” including patients with overlap myocarditis and myositis without myasthenia gravis. All other patients were classified as “not ICI myositis.”

Demographic information collected included age, sex, self‐identified race, smoking status, and cancer diagnoses at the time of first date of ICI administration, which was defined as the index date. Individual ICI medications were recorded, and each agent further was classified as PD‐1, PD‐L1, and CTLA‐4 inhibitors. Combination ICI therapy was defined as the delivery of both ipilimumab and nivolumab or durvalumab and tremelimumab concurrently.

### Statistical analyses

For Table 1, the continuous variable age was compared using analysis of variance and the categorical variables compared using the chi‐square test. The primary outcome was all‐cause mortality. Crude mortality was calculated as the percentage of patients surviving from the time of the initial ICI infusion to six months, one year, and two years, respectively. Confidence intervals (CIs) for crude mortality were calculated using a binomial calculator and, for the median survival, using a Kaplan–Meier estimator. The median survival for each myositis group was the median duration above which an equal number of patients had died and below which a similar number had survived with longevity greater than the median. Median survival was compared using a log rank test. Crude hazard ratios and hazard ratios adjusted for age, race, smoking status, cancer type, and ICI class were determined from the Cox regression models from the time of initial ICI infusion to six‐month, one‐year, two‐year, and five‐year follow‐up. For the Cox regression models and the Kaplan–Meier survival analysis, patients were censored at five years if surviving five years after the initial ICI infusions. To reduce potential of overfitting the model, the variables used in the adjustment were evaluated as dichotomous variables: age, over 70 years; race, White versus non‐White; smoking status, ever versus never/unknown; cancer type, lung versus non‐lung; and ICI class PD‐1 versus other ICI classes. Sex was not included in the model because all patients with ICI myositis were male. Statistical analyses were performed using STATA version 18 (StataCorp, LLC, College Station, Texas).

**Table 1:** Baseline characteristics at time of first ICI infusion*

	ICI triad syndrome (n = 25)	Isolated ICI myositis (n = 43)	No ICI myositis (n = 29,228)	Three group comparison
Age at initial ICI infusion, mean ± SD, y	76.0 ± 7.3	70.8 ± 9.9	69.9 ± 8.5	0.0013
Male sex, n (%)	25 (100)	43 (100)	28,272 (96.7)	0.317
Race, n (%)				0.021
Black or African American	0 (0)	13 (30.2)	5,187 (17.8)	
White	25 (100)	29 (67.4)	22,934 (78.5)	
Other	0 (0)	1 (2.3)	1,107 (3.8)	
Smoking status, n (%)				0.332
Ever	12 (48)	19 (44.2)	15,875 (53.8)	
Never	4 (16)	10 (23.3)	4,000 (13.6)	
Unknown	9 (36)	14 (32.6)	9,618 (32.6)	
Cancer diagnosis, n (%)				0.0001
Gastrointestinal	2 (8.0)	5 (11.6)	3,133 (10.7)	
Genitourinary	11 (44.0)	7 (16.3)	3,996 (13.7)	
Lung	3 (12.0)	20 (46.5)	13,349 (45.7)	
Melanoma	4 (16.0)	7 (16.3)	2,578 (8.8)	
Other	5 (20.0)	4 (9.3)	6,171 (21.1)	
Initial ICI therapy, n (%)				0.443
ICI monotherapy				
PD‐1				
Cemiplimab	0 (0)	1 (2.3)	265 (0.9)	
Nivolumab	9 (36.0)	13 (30.2)	8,728 (29.9)	
Pembrolizumab	12 (48)	22 (51.2)	12,544 (44.0)	
Subtotal	21 (84)	36 (83.7)	21,537 (73.7)	
PD‐L1				
Atezolizumab	0 (0)	1 (2.3)	3,078 (10.8)	
Avelumab	1 (4.0)	1 (2.3)	135 (0.5)	
Durvalumab	1 (4.0)	3 (7.0)	2,784 (9.8)	
Subtotal	2 (8.0)	6 (14)	5,997 (20.5)	
CTLA‐4				
Ipilimumab	2 (8)	2 (4.7)	992 (3.4)	
Subtotal	2 (8)	2 (4.7)	992 (3.4)	
ICI combination therapy^a^				
Ipilimumab + nivolumab	0 (0)	0 (0)	699 (2.4)	
Durvalumab + tremelimumab	0 (0)	0 (0)	3 (0.01)	
Subtotal	0 (0)	0 (0)	702 (2.4)	

* ICI, immune checkpoint inhibitor.

^a^ Combination therapy: the simultaneous delivery of ipilimumab and nivolumab or durvalumab and tremelimumab at first ICI infusion.

### Regulatory compliance

This study was approved by the University of Utah Institutional Review Board (IRB), which also serves as the IRB for the VA Salt Lake City Health Care System.

## RESULTS

There were 29,296 patients in VHA treated with an ICI during the study period. Of these, 6,248 (21%) had a CK level measured within six months of ICI infusion (Figure 1). There were 974 patients with myositis documented in the EHR. After exclusion of patients with clear evidence of conditions other than ICI myositis and inclusion of all patients with 10 × ULN CK levels, there were 188 patients who underwent level two chart review. There were 120 patients who did not meet criteria for ICI myositis. The most common conditions seen in patients undergoing level two chart review who did not have ICI myositis were septic shock, cardiogenic shock, myocardial infraction, myositis associated with infections, and rhabdomyolysis. After medical record review, 68 patients (0.2%) were categorized as ICI myositis. Of the patients with ICI myositis, 43 patients were further categorized as isolated ICI myositis and 25 patients as ICI triad.

### Baseline characteristics

Patients with ICI triad were significantly older, with a mean age at first ICI infusion of 76.0 years versus 70.8 years in patients with isolated ICI myositis and 69.9 years in patients without myositis (*P* = 0.0013; Table 1). All patients with ICI myositis (both ICI triad and isolated ICI myositis) were male. Patients with ICI triad were more likely to be White than patients with isolated ICI myositis (100% vs 67.4%, *P* = 0.021). No significant differences were found between smoking statuses. There were no differences in demographics between patients with isolated ICI myositis and patients treated with ICI who did not develop myositis.

### ICI treatment

The initial ICIs prescribed were atezolizumab, avelumab, cemiplimab, durvalumab, ipilimumab, nivolumab, pembrolizumab, and tremelimumab (Table 1). The most common ICI class was PD‐1 inhibitors, composing 73.7% of total ICIs prescribed, followed by PD‐L1 inhibitors, which made up 20.5% of all ICIs (Table 1). There were no significant differences between cancer immunotherapy class or drug and ICI myositis status. Combination therapy, defined as concurrent administration of two different ICIs at time of initial therapy, was not the initial therapy in any patients who developed isolated ICI myositis or ICI triad syndrome.

### Cancer history

The most common cancers were lung, genitourinary, melanoma, and gastrointestinal. Patients with ICI triad had more genitourinary cancers (44.0% vs 16.3%) and fewer lung cancers (12.0% vs 46.5%) when compared to patients with isolated ICI myositis (*P* = 0.0001).

### Laboratory outcomes

Patients with ICI triad had a significantly higher and earlier onset of CK elevation when compared to isolated ICI myositis. The first point that CK was measured after ICI therapy in patients with ICI triad was earlier than in patients with isolated myositis (29.3 vs 49.3 days, *P* = 0.005). The initial CK and CK‐MB levels measured following ICI therapy were significantly higher in patients with ICI triad (5,064 vs 3,457 IU/L, *P* = 0.0001, and 152 vs 110 IU/L, *P* = 0.0001, respectively). The time to peak CK measurement was significantly shorter in patients with ICI triad compared to isolated ICI myositis at 29.1 days versus 53.0 days (*P* = 0.04). Similarly, the peak CK level attained after ICI was delivered was higher in patients with ICI triad (5,204 vs 4,416, *P* = 0.0001). Myositis antibodies were rarely ordered and largely negative among patients with ICI myositis.

Skeletal muscle biopsy was obtained in three patients with ICI triad. Two of the three demonstrated acute necrotizing myopathy. Immunostaining revealed a few endomysial T cells, no significant B cells, and prominently perimysial with scattered endomysial histiocytes. The third showed no evidence of inflammatory or toxic myopathy.

### Mortality outcomes

During the six months after initial ICI infusion, patients developing ICI triad syndrome had median 60% (95% CI 38.72%–78.96%) mortality, which was higher than the 30.2% (17.2%–46.1%) mortality seen in patients developing isolated ICI myositis and 31.5% (31.0%–32.0%) mortality in patients without ICI myositis (Tables 2 and 3). Mortality was also higher in the ICI triad group at one year and two years of observation in comparison to the isolated ICI myositis and no ICI myositis groups. These differences were not statistically significant; however, interpretation is limited due to the increasingly low sample sizes at each time point. The median survival time was 2.5 (1.7–18.5) months in patients with ICI triad syndrome in comparison to the median survival of 20.4 (6.2–60) months in patients with isolated ICI myositis and 12.6 (12.3–12.9) months in patients without myositis.

**Table 2:** All‐cause mortality following first ICI*

	ICI triad syndrome (n = 25)	Isolated ICI myositis (n = 43)	No ICI myositis (n = 29,228)	Three group comparison^a^
6‐mo all‐cause mortality, % (95% CI)	60 (38.7–78.9)	30.2 (17.2–46.1)	31.5 (31.0–32.0)	0.013
1‐y all‐cause mortality, % (95% CI)	64 (42.5–82.0)	41.9 (27.0–57.9)	48.9 (48.3–49.4)	0.216
2‐y all‐cause mortality, % (95% CI)	72 (50.6–87.9)	53.5 (37.7–68.8)	65.8 (65.3–66.4)	0.211
Median 50% survival, months (95% CI)^b^	2.5 (1.7–18.5)	20.4 (6.2–60.0)	12.6 (12.3–12.9)	0.1651

* CI, confidence interval; ICI, immune checkpoint inhibitor; IQR, interquartile range.

^a^ Three group comparisons by chi‐square or all‐cause mortality and log rank test for median survival.

^b^ Median months at 50% mortality and 95% CIs.

**Table 3:** All‐cause mortality hazard ratios following first immune checkpoint inhibitor*

	ICI triad vs isolated ICI myositis	ICI triad vs no ICI myositis	Isolated ICI myositis vs no ICI myositis	ICI triad vs isolated ICI myositis^a^ *P* value	ICI triad vs no ICI myositis^a^ *P* value	Isolated ICI myositis vs no ICI myositis^a^ *P* value
Hazard ratio—Unadjusted (95% CI)						
6 mo	2.37 (1.12–5.00)	2.61 (1.57–4.33)	0.98 (0.57–1.70)	0.023	0.0001	0.957
1 y	1.78 (0.98–3.80)	1.86 (1.14–3.03)	0.85 (0.54–1.35)	0.057	0.013	0.492
2 y	1.78 (0.96–3.32)	1.52 (0.96–2.41)	0.76 (0.50–1.14)	0.068	0.076	0.183
5 y	1.51 (0.86–2.65)	1.29 (0.83–2.00)	0.66 (0.38–1.17)	0.156	0.256	0.156
Hazard ratio—adjusted^b^ (95% CI)						
6 mo	1.75 (0.77–3.98)	2.51 (1.51–4.16)	0.97 (0.56–1.66)	0.186	0.0001	0.904
1 y	1.79 (1.10–2.92)	1.79 (1.10–2.92)	0.84 (0.53–1.33)	0.020	0.020	0.46
2 y	1.46 (0.92–2.33)	1.47 (0.92–2.33)	0.75 (0.50–1.13)	0.104	0.104	0.166
5 y	1.20 (0.62–2.32)	1.22 (0.77–1.91)	0.74 (0.51–1.06)	0.588	0.396	0.100

* CI, confidence interval; ICI, immune checkpoint inhibitor.

^a^ Three group comparisons by chi‐square or all‐cause mortality and log rank test for median survival.

^b^ Adjusted for age, race, smoking status, cancer type, and class of initial ICI infusion.

**Figure 2:**
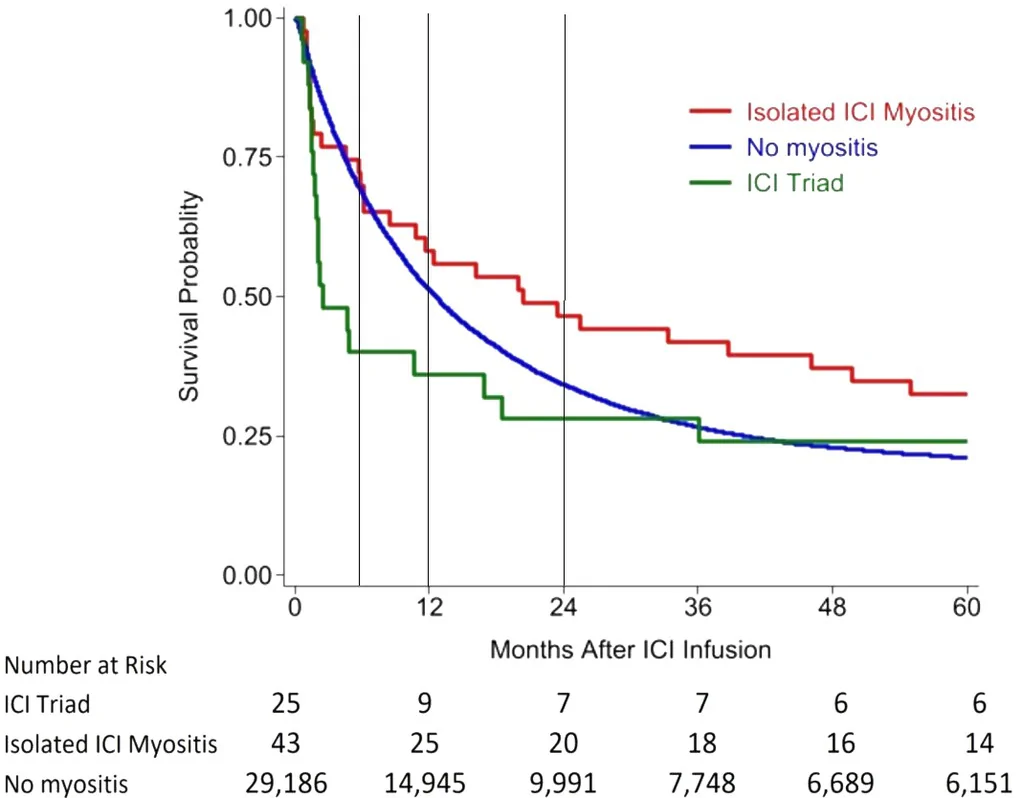
Kaplan–Meier curve demonstrating increased mortality of patients with ICI triad. ICI, immune checkpoint inhibitor.

The Kaplan–Meier survival curve for patients with ICI triad, patients with isolated ICI myositis, and patients without myositis over the five years following initial ICI infusion shows a high mortality in patients with ICI triad during the first year with a subsequent merging of mortality of the three groups over the following years (Figure 2). During the first six months after initial ICI infusion, Cox regression models showed higher hazard ratios for the patients with ICI triad group in comparison to patients with isolated ICI myositis and without myositis at 2.37 (95% CI 1.12–5.00) and 2.61 (95% CI 1.57–4.33) (Tables 2 and 3). After one year of follow‐up, the mortality of the ICI triad group remained high in comparison to isolated ICI myositis; however, results were not statistically significant. After adjusting the Cox regression model for age, race, smoking status, cancer group, and initial ICI class, the hazard ratios over six months comparing patients with ICI triad to patients with isolated ICI myositis and no ICI myositis were 1.75 (95% CI 0.77–3.98) and 2.51 (95% CI 1.51–4.16). Because all patients with ICI myositis were male, the Cox regressions could not be adjusted for sex; however, an analysis limited to only male patients in all groups showed similar results to the full population (data not shown).

## DISCUSSION

This report is the first nationwide multicenter cohort study evaluating ICI‐associated myositis outcomes and represents one of the largest studies to date on ICI myositis triad. The size of our cohort afforded us a unique opportunity to compare ICI‐associated myositis to the general population treated with ICI. These data confirm that although ICI‐associated myositis is a significant irAE, the highest mortality is in patients with ICI myositis triad syndrome. We found that patients with ICI myositis triad had substantially increased mortality during the six months after the first infusion, though it is unclear if there is an increased risk for those that survive past this first six‐month period.

Although patients with ICI myositis triad in our cohort had a higher mortality than patients without ICI myositis, patients with isolated ICI myositis had a similar survival compared to patients who did not develop myositis. This result may be partially due to the small sample size in the isolated ICI myositis group. Alternately, Saygin et al asserted that, in patients with ICI myositis, the most common cause of death is cancer progression; in overlap patients, it is the irAE.^11^ If true, that would suggest that patients who survive ICI myositis may experience improved cancer outcomes. Although we were not able to capture progression of disease in our study, our results support previous findings that the cancer response rate may be higher in patients with irAEs.^12^, ^13^, ^14^

This study also demonstrated the early onset of ICI myositis triad and isolated ICI myositis after ICI initiation with the time to peak CK measurement of 29.1 days and 53.0 days, respectively. This is consistent with other studies demonstrating myositis as one of the earliest developing irAEs, with reports of a median onset of 31 days.^15^, ^16^, ^17^ The acute nature of this irAE development is important for providers and patients to be aware of to increase early diagnosis and intervention.

Prior studies have established ICI myositis triad syndrome as a rare and often lethal adverse effect of cancer immunotherapy.^2^, ^4^, ^13^, ^18^ Although most patients will develop at least one adverse event secondary to ICI therapy, death from irAEs only represents 0.86% of all treated patients.^14^, ^19^ The World Health Organization pharmacovigilance surveys measured the mortality of ICI myositis at 21.2%.^2^ ICI triad has mortality ranging from 42% to 51% in retrospective cohort studies.^5^, ^6^ Our study further confirms that the prevalence of irAE myositis is low (0.23%), but mortality, specifically with ICI myositis triad, is high (60%). Due to the high mortality, early evaluation for ICI myositis triad should be obtained and supportive care provided.

Patients with ICI triad syndrome were significantly older than patients with isolated ICI myositis, with a median age of 76.1 versus 70.9 years at time of initial ICI infusion. Although rates of low‐grade immune toxicities are similar regardless of age, multiple studies have found a higher incidence of common terminology criteria for adverse events grade 3 and higher irAEs in patients 65 years and older.^17^, ^20^, ^21^

Additionally, we found a potential signal for genitourinary cancers for the development of ICI triad syndrome that requires future investigation. Genitourinary cancers were twice as common in ICI triad compared to isolated ICI myositis. Multiple case reports describe patients with ICI triad with genitourinary cancers.^22^, ^23^, ^24^, ^25^ The patient from the study by Botta et al had elevated activated CD8 T cells and the presence of HLA‐DRB1*03/*04 haplotype. If this finding is confirmed in other studies, it could support the theory that ICI triad is secondary to the inappropriate activation of T cells against a shared epitope between tumor cells and healthy muscle.^26^ Perhaps urothelial cancer, which is enriched in smooth muscle, increases the chances of immune dysregulation targeting normal myocytes.

Strengths of our study included the availability of the VA standardized national EHR in the largest health care system in the US. We were able to access these EHRs and data collection systems across facilities nationwide. In addition, we were able to compare those with ICI triad syndrome, those with isolated ICI myositis, and those with ICI who did not develop myositis, a novel investigation in this arena.

Limitations of this study include the retrospective study design. Although our definition of ICI myositis was specific, we may have missed cases in which CK was not measured or myositis was not noted in the EHR. Similarly, we may not have captured cases that were labeled as irAE myocarditis or myasthenia gravis, thereby overshadowing a diagnosis of ICI myositis. Veterans who had ICI treatment within VHA may also have developed and been diagnosed with ICI‐associated myositis outside VHA and not captured in our analysis. Additionally, our patients were White male veterans, which limits generalizability. Although this report is one the largest reports of ICI‐associated myositis to date, the relatively small numbers limit the detection of risk factors. Although efforts were made to adjust for potential confounding factors, we recognized the potential for unidentified residual confounders.

Our study adds to the knowledge of ICI‐induced adverse events by describing and comparing ICI triad and isolated ICI myositis subsets in a large national population. ICI triad syndrome is associated with worse overall survival and median survival times in comparison to patients with isolated ICI myositis and patients treated with ICI without ICI myositis. In contrast, patients with isolated ICI myositis have similar outcomes to patients treated with ICI who did not develop myositis. These data provide useful information to clinicians faced with these irAEs as they communicate expected outcomes. These data also highlight the need for early recognition and treatment of these disease entities.

## AUTHOR CONTRIBUTIONS

All authors contributed to at least one of the following manuscript preparation roles: conceptualization AND/OR methodology, software, investigation, formal analysis, data curation, visualization, and validation AND drafting or reviewing/editing the final draft. As corresponding author, Dr Rubino confirms that all authors have provided the final approval of the version to be published and takes responsibility for the affirmations regarding article submission (eg, not under consideration by another journal), the integrity of the data presented, and the statements regarding compliance with institutional review board/Declaration of Helsinki requirements.

## Supporting information


**Disclosure Form**:
